# Social Capital as a Determinant of Self-Rated Health in Women of Reproductive Age: A Population-Based Study

**DOI:** 10.5539/gjhs.v8n2p273

**Published:** 2015-07-20

**Authors:** Azam Baheiraei, Fatemeh Bakouei, Sareh Bakouei, Narges Eskandari, Hoda Ahmari Tehran

**Affiliations:** 1 Community Based Participatory Research Center, Iranian Institute for Reduction of High-Risk Behaviors, Tehran University of Medical Sciences, Tehran, Iran; 2 Department of Midwifery, Babol University of Medical Sciences, Babol, Iran; 3 Faculty of Nursing and Midwifery, Qom University of Medical Sciences, Qom, Iran; 4 Reproductive Health, Schools of Midwifery Shahid Beheshti University of Medical Sciences; 5 Faculty of Nursing and Midwifery, Qom University of Medical Sciences, Qom, Iran

**Keywords:** population-based study, reproductive age, self-rated health, social capital, women

## Abstract

**Introduction:**

Recognition of the factors related to women’s health is necessary. Evidence is available that the social structure including social capital plays an important role in the shaping people’s health. The aim of the current study was to investigate the association between self-rated health and social capital in women of reproductive age.

**Methods:**

This study is a population-based cross-sectional survey on 770 women of reproductive age, residing in any one of the 22 municipality areas across Tehran (capital of Iran) with the multi stage sampling technique. Self-rated health (Dependent variable), social capital (Independent variable) and covariates were studied. Analysis of data was done by one-way ANOVA test and multiple linear regressions.

**Results:**

Depending on logistic regression analyses, the significant associations were found between self-rated health and age, educational level, crowding index, sufficiency of income for expenses and social cohesion. Data show that women with higher score in social cohesion as an outcome dimension of social capital have better self-rated health (PV = 0.001).

**Conclusion:**

Given the findings of this study, the dimensions of social capital manifestations (groups and networks, trust and solidarity, collective action and cooperation) can potentially lead to the dimensions of social capital outcomes (social cohesion and inclusion, and empowerment and political action). Following that, social cohesion as a dimension of social capital outcomes has positively relationship with self- rated health after controlling covariates. Therefore, it is required to focus on the social capital role on health promotion and health policies.

## 1. Introduction

The individual or collective health is undoubtedly one of the most important aspects of human life issues. All human can be active completely, if they feel themselves healthy and also society deems them healthy (Kolander, 1999). Half the world’s population are the women who play an important role in the cultural and educational structure, preservation and promotion of the family and the subsequent impact on society. In general, women’s health is an important foundation for the development of public health (Barooti, Sadeghi, Karimi-Zarchi, & Soltani, 2011). Therefore, health policies and programs should focus on the components affecting women’s health (Bustreo, Knaul, Bhadelia, Beard, & Araujo de Carvalho, 2012).

Recognition of the factors related to women’s health is necessary to develop strategies to reduce the incidence of some diseases and mortality, and to increase the quality of life (Cabeza, Esteva, Pujol, Thomas, & Sánchez-Contador, 2007). James et al. state that to improve women’s health all factors should be measured (James et al., 2009). Evidence is available that social structure including social capital plays an important role in shaping people’s health (Poortinga, 2006).

Social capital like the other forms of capital (human, financial, physical), also provides benefits for people (Coleman, 1990). The hypothesized mechanisms of social capital in relationto health, including better accessto information, helping to improve decision-making related to health (such as diet, exercise, or selection of adoctorora hospital) and impact onsocial norms (for example, encouraging peopletolose weight, quit smoking, or vaccination) (Scheffler & Brown, 2008).

The studies have shown that the variety of health-related outcomes such asself-rated health (Kim, Subramanian, Gortmaker, & Kawachi, 2006), cardiovascular disease (Sundquist, Johansson, Yang, & Sundquist, 2006), mental health (Lofors & Sundquist, 2007) and health behaviors (Brown, Scheffler, Seo, & Reed, 2006; Kim et al., 2006) are associated with social capital. However, someother studies have found no correlation (Blakely et al., 2006; Mohan, Twigg, Barnard, & Jones, 2005). In summary, the findings ofthe researchto evaluate the relationship between social capital and health are different in terms of the concept of social capital indicators, level of analysis, social context and demographic variables that constitute the framework of the study.

Ina meta-analysis conducted in 2012, in most articles reviewed conductedin western countriesand the research was very little in Asiancenters, however shown that social capitalis not always associated with beneficial effects on health outcomes (Murayama, Fujiwara, & Kawachi, 2012). Inhigh-incomecountries, highersocial capitalis associated with better health, but the evidenceis little in low and middle-income countries (Riumallo-Herl, Kawachi, & Avendano, 2014). About the issues that are social context based, it cannot rely on the results of other countries or the meta-analysis studies in the policy making of our country.

One issue inthe social sciencesis thebestwayto measuresocial capital. In this study Integrated Questionnaire for the Measurement of Social Capital (SC-IQ) designed by World Bank was used; because itincludesall the social capitalindicators that are in the studies (Grootaert, Narayan, Jones, &Woolcock, 2004).

This research is essential because of the role of women in promoting family health, and their priority in the reproductive health programs in a country. Since most of the women population in Iran are women of reproductive age (Statistical Center of Iran, 2011), the aim of the current study was to investigate the association between self-rated health and social capital in women of reproductive age.

## 2. Methods

### 2.1 Setting and Data Collection

This study is a population-based cross-sectional survey on women of reproductive age (15-49 years old), residing in Tehran (capital of Iran). The sample size in the current study was 770 people with P = 50 %, d = 4 %, and considering design effect (df = 1.2 %). The sampling technique was multi stage inmunicipality areas of Tehran that each area was divided into a number of blocks with unequal population. In each area, the required number of blocks was randomly selected from the list of blocks. Next, using the systematic sampling method, the women ofreproductive age in households were interviewed. In case a woman declined to take part, the next door household was invited to participate in the study to complete the questionnaire. It must be noted that they were ensured about the confidentially of their information.

All participants were personally interviewed in their own house by the team of interviewers. Interviewers were trained by the research team on how to conduct standard interviews. To ensure reliability of the collected data, the interviewers were closely controlled and monitored. Study protocol was approved by the Ethics Committee. Following a detailed explanation, informed consent was obtained from all participating women.

### 2.2 Dependent Variable (Outcome Measure - Self-Rated Health)

Self-rated health (SRH) is one of the most used methods to assess the public health status of populations (Jia et al., 2014). It also serves as a validity predictor of mortality and morbidity in longitudinal studies (Hibino et al., 2012). SRH was measured with this question: ‘‘How would you describe your health at present?’’ On a five-point scale (excellent=1, very good=2, good=3, fair =4, poor=5). In the analysis, this variable was dichotomized into good (1= combining good, very good and excellent) and poor (0= combining fair and poor) health.

### 2.3 Independent Variable (Social Capital)

In this study, social capital was assessed by Social Capital Integrated Questionnaire. This questionnaire (SC-IQ) was designed by the World Bank in 2000 for measuring the social capital of developing countries. The questionnaire contains 6 dimensions: groups and networks, trust and solidarity, collective action and cooperation, information and communication, social cohesion and inclusion, and empowerment and political action (Grootaert et al., 2004). The validity and reliability of the questionnaire was measured by Nedjat et al. in Iran through forward-backward translation method. The mean score for the 5 dimensions except the dimension of information and communication (that was only for more detailed reviewing of obtaining information from the general public) ranged from 0 to 100, with a higher score indicating higher social capital. The Intra Class Correlation Coefficient (ICC) was higher than 0.7 in all the dimensions (ICC range: 0.75-0.89) (Nedjat, Majdzadeh, Kheiltash, Jamshidi, & Yazdani, 2013). According to the recommendations of instrument designers of the World Bank Organization, the researchers of this study assumed groups and networks, trust and cooperation as manifestations of social capital and social cohesion and empowerment and political action as outcomes of social capital. The scores were calculated for all aspects in a range of 0-100 (Grootaert et al., 2004).

### 2.4 Covariates

Other variables included in this analysis were socio-demographic variables: age, ethnicity, educational level, marital status, family size, occupation status, sufficiency of income for expenses, and crowding index.

### 2.5 Statistical Methods

Descriptive analysis and chi-squared tests were conducted to compare the differences between SRH and potential health-influencing factors (covariates). To avoid the effects of the confounding factors, and to predict effects of independent variables (dimensions of social capital) on SRH, the covariates with significant associations in the chi-squared test were entered into the multiple logistic regressions. Multivariate-adjustedodds ratios (OR) and 95% confidence intervals (CI) werecalculated from logistic regressions to examine the factorsthat influence SRH.

To investigate and confirm the relationship among the manifestation dimensions of social capital (groups and networks, trust and solidarity, collective action and cooperation) with the outcome dimensions of social capital (social cohesion and inclusion, and empowerment and political action), the linear regression analysis was used. For all analyses, statisticalsignificance was set at 0.05. Statistical analysis wasperformed using the Statistical Package for Social Sciences (SPSS 16).

## 3. Results

### 3.1 Participants Characteristics

In total, 770, 15- to 49-year-old women of reproductive age with mean age 33.9±9.3 years were interviewed. Majority of them were married (72.2%), housewives (62.2%), of Persian ethnicity (64.3%), and educated up to high school level (43.8%) (Table 1).

**Table 1:** The characteristics of the study sample (n=770)

variables	Number (%)
**Age groups**	
15-25 years	165 (21.5)
26-35 years	266 (34.5)
36-45 years	224 (29.1)
46-49 years	115 (14.9)
**Marital status**	
Married	556 (72.2)
Divorced/ Widowed	31 (4)
Single	183 (23.8)
**Ethnicity**	
Fars	487 (64.3)
Azari	179 (23.2)
Others	91 (13.5)
**Educational level**	
Illiterate/ Primary (years 1-5)	50 (6.5)
Intermediate (years 6-8)	83 (10.8)
High school (years 9-12)	337 (43.8)
University	300 (38.9)
**Occupation status**	
Housewife	479 (62.2)
Employed	190 (24.7)
Student	101 (13.1)
**Sufficiency of income for expenses**	
Absolutely not	337 (44.1)
To some extent	240 (31.4)
Completely	188 (24.5)
**Crowding index**	
Low	195 (27.5)
Average	365 (51.6)
High	148 (20.9)
**Family Size**	
Less than four	321 (42.3)
Four to five	384 (50.7)
More than five	53 (7.0)

### 3.2 Bivariate Analyses: The Major Difference between Covariates and SRH

Table 2 presents the differences in the distribution of all the covariates between the two groups (Poor and good health). Table 2 shows the proportion of women who reported good SRH decreased significantly with age (P = 0.001). The proportion of married individuals who reported good SRH was lower than that of the unmarried individuals (P = 0.001). Also, the highest proportion related to good SRH was in women with university educational status, low crowding index and sufficiency of income for expenses completely (P = 0.001, P = 0.015, P = 0.001)

**Table 2:** Respondents’ characteristics in association with Self-Rated Health through chi-square test

Covariates	Self-Rated Health: Number (%)		

	Poor	Good	chi-square p.V
**Age groups**			
15-25 years	34 (20.7)	130 (79.3)	0.001
26-35 years	64 (24.1)	202 (75.9)	
36-45 years	90 (40.4)	133 (59.6)	
46-49 years	61 (53.5)	53 (46.5)	
**Marital status**			
Married	198 (35.7)	356 (64.3)	0.001
Divorced/ Widowed	14 (45.2)	17 (54.8)	
Single	37 (20.3)	145 (79.7)	
**Ethnicity**			
Fars	152 (31.3)	333 (68.7)	0.057
Azari	70 (39.1)	109 (60.9)	
Others	27 (26.2)	76 (73.8)	
**Educational level**			
Illiterate/ Primary (years 1-5)	28 (57.1)	21 (42.9)	0.001
Intermediate (years 6-8)	37 (44.6)	46 (55.4)	
High school (years 9-12)	121 (36)	215 (64)	
University	63 (21.1)	236 (78.9)	
**Occupation status**			
Housewife	179 (37.5)	298 (62.5)	0.001
Employed	55 (28.9)	135 (71.1)	
Student	15 (15)	85 (85)	
**Sufficiency of income for expenses**			
Absolutely not	135 (40.2)	201 (59.8)	0.001
To some extent	67 (27.9)	173 (72.1)	
Completely	45 (24.2)	141 (75.8)	
**Crowding index**			
Low	48 (24.6)	147 (75.4)	0.015
Average	124 (34.2)	239 (65.8)	
High	57 (38.5)	91 (61.5)	
**Family Size**			
Less than four	92 (28.7)	229 (71.3)	0.119
Four to five	137 (36)	244 (64)	
More than five	18 (34)	35 (66)	

Total	249 (32.5)	518 (67.5)

### 3.3 Multivariate Analyses: Effect Social Capital and Covariates on SRH

Logistic regression analyses were used to identify thepotential factors (social capital and covariates) that influenced SRH (Table 3).This table shows only variables which remained as significant factors after adjusting other variables in regression analysis with SRH. As can be seen from Table 3, significant associations were found between SRH and the following variables; age, educational level, crowding index, sufficiency of income for expenses and social cohesion.Data shows that women with higher score in social cohesion as an outcome dimension of social capital are significantly associated positively with better SRH.

**Table 3:** Multi-variable (adjusted) logistic regression: the association of self-rated health with social capital and covariates, (odds ratio (OR) and 95% CI)

Predictor	self-rated health		

	OR	95% CI	p-value
**Age** a	0.94	0.92 - 0.96	0.001
**Crowding Index**			
Low	1.00	-	
Average	0.65	0.43 - 1.006	
High	0.48	0.28 - 0.80	0.006
**Educational level**			
Illiterate/ Primary (years 1-5)	1.00	-	
Intermediate (years 6-8)	1.49	0.68 - 3.26	
High school (years 9-12)	1.80	0.91- 3.57	
University	2.98	1.46 - 6.08	0.003
**Sufficiency of income for expenses**			
Completely	1.00	-	
Absolutely not	0.61	0.38 - 0.98	0.040
To some extent	0.83	0.50 - 1.37	
**Social cohesion** a	1.03	1.02 - 1.05	0.001

a: Quantitative variable

The variables not shown in the table were not significantly associated.

Moreover, the linear regression analysis confirmed that the three manifestation dimensions are significantly related to two outcome dimensions of social capital. Besides, as shown in Table 3, after importing all the dimensions of manifestation and outcome of social capital to the logistic regression model and considering SRH as the dependent variable, only the dimension of outcome, social cohesion remained in the regression model.

## 4. Discussion

The objective of the current study was to determine a possible association between social capital and self-rated health among women of reproductive age. In this study, the highest percent age participants (67.5%) had good SRH.In the final analysis, the logistic regression model, data have presented a significant adjusted relationship between social cohesion and SRH after controlling covariates. On the other hand, the findings of this study also supported World Bank claim in social capital integrated questionnaire used in the present study on the relationship between dimensions of manifestation and outcome. It means that, women with higher scores in groups and networks, trust, and participation achieved higher scores in social cohesion and inclusion and empowerment and political action. This finding is similar with other studies (Berry, 2008; Berry & Welsh, 2010). As a result, it is required not only to focus on communications and groups of people, but also on the value of these communications as social capital resources.

According to the mentioned findings, authors can conclude that social capital is a potential factor related health.In another study by Chappell and Funk also reported that although the direct relationship was not found between group networks, participation in community activities and trust with any of the health aspects, their interaction test confirmed the integrated utility of social capital (Chappell & Funk, 2010). The other studies also showed that at least one of dimensions of social capital was related to health (Verhaeghe et al., 2012; Riumallo-Herl, Kawachi, & Avendano, 2014).

Eriksson (2010) found that the cognitive dimensions of social capital such as trust and a sense of security (especially feeling of safe to walk alone at night) is rather moreclosely related to health than the structural dimensions of social capital. In the current study, also the questions relating to security (such as, feeling of safe to walk alone at night; feeling of safe in the neighborhood and home) were in the social cohesion dimension which were significantly associated with SRH.

According to the logistic regression model it was also observed that the educational level, crowding index and sufficiency of income for expenses (as the economic variables) were significantly associated with SRH. The other studies also showed the impact of human capital, financial capital and social capital on health (Ziersch, 2005; Poortinga, 2006).

## 5. Conclusion

Given the findings of this study, the dimensions of social capital manifestations (groups and networks, trust and solidarity, collective action and cooperation) can potentially lead to the dimensions of social capital outcomes (social cohesion and inclusion, and empowerment and political action). Following that, social cohesionas a dimension of social capital outcomeshas positively relationship withself- rated health after controlling covariates. Therefore, it is required to focus on the social capital role on health promotion and health policies.

## References

[ref1] Barooti E, Sadeghi N, Karimi-Zarchi M, Soltani H. R (2011). New results regarding trends in Iranian women’s health and a comparison with WHO data. Clin Exp Obstet Gynecol.

[ref2] Berry H. L (2008). Social capital elite, excluded participators, busy working parents and aging, participating less: types of community participators and their mental health. Soc Psychiatry PsychiatrEpidemiol.

[ref3] Berry H. L, Welsh J. A (2010). Social capital and health in Australia: An overview from the household, income and labor dynamics in Australia survey. Soc Sci Med.

[ref4] Blakely T, Atkinson J, Ivory V, Collings S, Wilton J, Howden-Chapman P (2006). No association of neighborhood volunteerism with mortality in New Zealand: A national multilevel cohort study. Int J Epidemiol.

[ref5] Brown T. T, Scheffler R. M, Seo S, Reed M (2006). The empirical relationship between community social capital and the demand for cigarettes. Health Econ.

[ref6] Bustreo F, Knaul F. M, Bhadelia A, Beard J, Araujo de Carvalho I (2012). Women’s health beyond reproduction: Meeting the challenges. Bull World Health Organ.

[ref7] Cabeza E, Esteva M, Pujol A, Thomas V, Sánchez-Contador C (2007). Social disparities in breast and cervical cancer preventive practices. Eur J Cancer Prev.

[ref8] Coleman J. S (1990). Foundations of social theory.

[ref9] Grootaert C, Narayan D, Jones V. N, Woolcock M (2004). Measuring social capital: An integrated questionnaire.

[ref10] Hibino Y, Takaki J, Ogino K, Kambayashi Y, Hitomi Y, Shibata A, Nakamura H (2012). The relationship between social capital and self-rated health in a Japanese population: A multilevel analysis. Environ Health Prev Med.

[ref11] James C. V, Salganicoff A, Thomas M, Ranji U, Lillie-Blanton M, Wyn R (2009). Putting women’s health care disparities on the map: Examining racial and ethnic disparities at the state level.

[ref12] Jia Y, Gao J, Dai J, Zheng P, Wu X, Li G, Fu H (2014). Difference of the associations between self-rated health and demographic characteristics, lifestyle, and psychosocial work environment between two types of Chinese worksite. BMC Public Health.

[ref13] Kim D, Subramanian S. V, Gortmaker S. L, Kawachi I (2006). US state- and county-level social capital in relation to obesity and physical inactivity: A multilevel, multivariable analysis. Soc Sci Med.

[ref14] Kolander Ch. A (1999). Contemporary Women’s Health.

[ref15] Lofors J, Sundquist K (2007). Low-linking social capital as a predictor of mental disorders: A cohort study of 4.5 million Swedes. Soc Sci Med.

[ref16] Mohan J, Twigg E, Barnard S, Jones K (2005). Social capital, geography and health: A small-area analysis for England. Soc Sci Med.

[ref17] Murayama H, Fujiwara Y, Kawachi I (2012). Social capital and health: A review of prospective multilevel studies. J Epidemiol.

[ref18] Nedjat S, Majdzadeh R, Kheiltash A, Jamshidi E, Yazdani S (2013). Social Capital in Association with Socioeconomic Variables in Iran. Soc Indic Res.

[ref19] Poortinga W (2006). Do health behaviors mediate the association between social capital and health?. Prev Med.

[ref20] Riumallo-Herl C. J. R, Kawachi I, Avendano M (2014). Social capital, mental health and biomarkers in Chile: Assessing the effects of social capital in a middle-income country. Soc Sci Med.

[ref21] Scheffler R. M, Brown T. T (2008). Social capital, economics, and health: New evidence. Health Economics, Policy and Law.

[ref22] Sundquist J, Johansson S. E, Yang M, Sundquist K (2006). Low linking social capital as a predictor of coronary heart disease in Sweden: A cohort study of 2.8 million people. Soc Sci Med.

[ref23] Ziersch A. M (2005). Health implications of access to social capital: Findings from an Australian study. Soc Sci Med.

